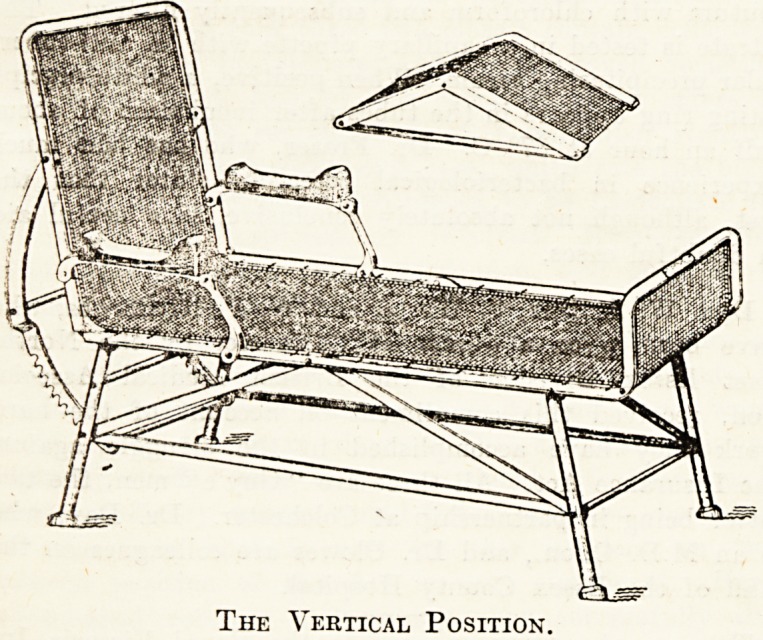# A Convenient Method of Moving Patients

**Published:** 1914-04-04

**Authors:** 


### THE LLOYD SMITH SANATORIUM CHAIR.

### A Convenient Method of Moving Patients.

We append below two illustrations of the Lloyd Smith sanatorium chair in use at Benenden Sanatorium, which is referred to in our Report on that institution on page 15 of the present issue. The design of this chair is based on that of a German model, which has been improved upon in construction and in convenience of adjustment by Dr. D. Lloyd Smith, medical superintendent of the Manchester Hospital for Consumption, Bowden, Cheshire. It consists essentially of a rectangular iron framework, and is entirely constructed, except

The Horizontal Position.

the wooden arm rests and cast-iron feet, of wrought and malleable iron. Rigidity of the structure is ensured by a system of tie-rods and stays. An extra strong seating of galvanised woven-wire, forming a sort of spring mattress, is tightly stretched in the rectangular frame, which is given a short upward bend at the foot, while at about one-third of its length from the head, the frame is hinged, so as to form an adjustable back rest, which can be raised to the vertical, lowered to the

The Vertical Position.

horizontal, or fixed at any angle between the two. Arm rests are provided, and are hinged in such a way as not to interfere with the movement of the back rest. The knee rest or pillow can be obtained in two patterns, rigid or adjustable, the latter form being constructed in a manner which allows the angle between the two slopes to be varied to suit the comfort of patients of different heights. Thick wool felt pads, the length and width of the chair, are supplied in different qualities and colours which will stand sterilising. In place of the cast-iron feet, rubber-tyred castors can be fitted, enabling a patient reclining upon the chair to be easily and silently moved about the wards or into verandahs.

A convenient mode of conveying patients up and down stairs, or to and from the operation theatre, is provided by the use of a " wood-carrying frame " in conjunction with the chair. This consists essentially of two long poles with handles at each end, and the carrying frame is so constructed as to fit firmly and safely between the legs of the chair, immediately under the mattress or seating. The weight of the chair is only 63 lbs., so that two attendants can easily and comfortably carry a patient to any part of the buildings or grounds. The length of the chair is 6 feet 6 inches when flat, and the width over all is 2 feet ½ inch. The materials of which they are made are designed to makes these chairs practically indestructible and much more durable than those of the ordinary cane lounge chairs in general use. Messrs. Isaac Charlton and Co., Manchester, are the makers, and in addition to the National Sanatorium, Benenden, Kent, the chairs are in use at the Manchester Hospital for Consumption, the Manchester Fever Hospital, and the Liverpool Sanatorium.

## Figures and Tables

**Figure f1:**
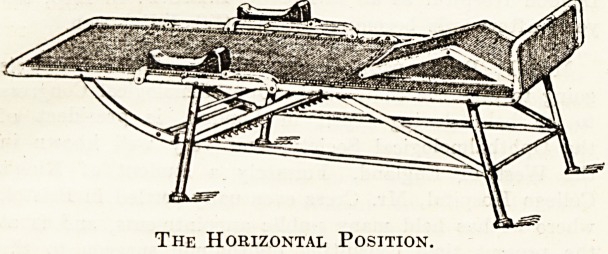


**Figure f2:**